# Sources of evidence

**DOI:** 10.1590/S1516-31802008000200002

**Published:** 2008-03-06

It is clearly more efficient for us to update ourselves through good sources of evidence than to try to keep track of the roughly two million articles that are published in the field of healthcare every year.

Excellent sources of evidence do exist, as our readers are already tired of hearing about. This will increasingly be the role of good medical journals. Just so that we do not lose the habit, we will again recommend here the Cochrane Library (http://cochrane.bvsalud.org/), ACP Journal Club (http://www.acpjc.org/) and the Bulletin of the Agency for Healthcare Research and Quality (AHRQ) of the U.S. Department of Health and Human Services (http://www.ahrq.gov/research/resact.htm).

Worldwide, well-prepared health ministries are in the habit of ordering systematic reviews and evidence-based healthcare technology assessments from specific centers, generally based on reviews using the Cochrane methodology. The Brazilian Ministry of Health, through its Department of Technological Assessment, recently received 27 reviews (Table 1) that had been prepared by teams at the Brazilian Cochrane Center. Some of them made it possible to prevent expenditure of around R$1.2 billion over the three years preceding the publication of the results by the Cochrane Library and in a major journal, which were shown to be the same as those prepared by the team at the Brazilian Cochrane Center.^1^

**Table 1.** List of reviews prepared by teams at the Brazilian Cochrane Center for the Ministry of Health

01. Acupuncture for headache
02. Acupuncture for lateral epicondylitis
03. Acupuncture for low back pain
04. Acupuncture for carpal tunnel syndrome
05. Adalimumab for treating rheumatoid arthritis
06. Ferrara's ring for treating keratoconus
07. Intragastric balloons for obese individuals
08. Reused versus new electrophysiology catheters
09. Roux-en-Y gastric derivation for surgically treating morbid obesity
10. Drotrecogin for treating severe sepsis
11. Efalizumab for treating psoriasis
12. Embolization for treating uterine myoma
13. Comparative study on treatments for Parkinson's disease: deep cerebral stimulation using electrodes versus neuroablative procedures
14. Imatinib for chronic myeloid leukemia
15. Imatinib for treating gastrointestinal tumors
16. Skin replacement materials for treating burns
17. Nucleoplasty for treating disc hernias
18. Suburethral suspension operations for urinary incontinence in women
19. Stents covered with rapamycin or paclitaxel versus conventional stents
20. Mechanical suture versus manual suture in colorectal anastomoses
21. Intersomatic fusion techniques at single or double intervertebral level for treating cervical degenerative disc disease
22. Surgical treatment techniques for morbid obesity: Mason bands/gastroplasty
23. Surgical treatment techniques for morbid obesity: duodenal switch/Scopinaro
24. Photodynamic therapy for age-related neovascular macular degeneration
25. Teriparatide [recombinant human parathyroid hormone (1-34)] for treating osteoporosis in postmenopausal women
26. Cardiac resynchronization therapy: use of multisite pacemaker
27. Surgical treatments for epilepsy

An article prepared at the request of the AHRQ, by Duke University's Evidence-Based Practice Center, showed on a large sample of 15,000 cases that, in treating arterial hypertension, the effects of angiotensin-converting enzyme (ACE) inhibitors and angiotensin receptor inhibitors were very similar in clinical practice. The only difference found was in the prevalence of coughing: 1.7% of the patients who received ACE inhibitors versus 0.6% of those who received angiotensin receptor inhibitors. This report is available in full on the website http://effectivehealthcare.ahrq.gov/index.cfm.^2^

Even more interesting is an article published in the Archives of Pediatric and Adolescent Medicine, which showed that in the United States, Latinos hardly ever drink tap water and do not give tap water to their children. They almost always used bottled mineral water, like in Brazil.^3^ This takes away from their children the fluoride that is added to tap water and compromises the oral health of the population, if the fluoride is not adequately supplemented. The most interesting point in this is that the quality requirements for tap water are higher than are those for bottled mineral water. The situation is not expected to be any different in Brazil. In clinical practice, we have seen cases of diarrhea relating to water described as mineral water that is delivered to homes. And I ask: is there any proof of quality control over water sold in Brazil?

By carrying out a search using the terms “fluoride” and “boiled water” in databases like the Latin-American and Caribbean Health Sciences Literature (*Literatura Latino-americana e do Caribe em Ciências da Saúde*, Lilacs), Brazilian Dental Bibliography (*Bibliografia Brasileira de Odontolologia*, BBO) and Scirus, we find evidence that boiling tap water maintains or increases the fluoride concentration, which may be a good solution.^4,5^

One further point of great interest: the AHRQ has just released a DVD on how to build hospitals (or how to refurbish them, which is our eternal problem), to assist architects, engineers and physicians to build hospitals in which the plans incorporate principles of evidence-based hospital architecture.^6^ This is available on the website http://www.ahrq.gov/qual/transform.htm. We are getting there.
